# Homozygous loss-of-function variants in European cosmopolitan and isolate populations

**DOI:** 10.1093/hmg/ddv272

**Published:** 2015-07-14

**Authors:** Vera B. Kaiser, Victoria Svinti, James G. Prendergast, You-Ying Chau, Archie Campbell, Inga Patarcic, Inês Barroso, Peter K. Joshi, Nicholas D. Hastie, Ana Miljkovic, Martin S. Taylor, Generation Scotland, UK10K, Stefan Enroth, Yasin Memari, Anja Kolb-Kokocinski, Alan F. Wright, Ulf Gyllensten, Richard Durbin, Igor Rudan, Harry Campbell, Ozren Polašek, Åsa Johansson, Sascha Sauer, David J. Porteous, Ross M. Fraser, Camilla Drake, Veronique Vitart, Caroline Hayward, Colin A. Semple, James F. Wilson

**Affiliations:** 1 MRC Human GeneticsUnit, MRC Institute of Genetics and Molecular Medicine and; 2 Centre for Genomicand Experimental Medicine, Institute of Genetics and Molecular Medicine, University of Edinburgh, Western General Hospital, Crewe Road, EdinburghEH4 2XU, UK,; 3 The Roslin Institute, The University of Edinburgh, Easter Bush, MidlothianEH25 9RG, UK,; 4 Medical School, University of Split, Soltanska 2, Split21000, Croatia,; 5 Wellcome Trust Sanger Institute, Hinxton, CambridgeCB10 1SA, UK,; 6 Usher Institute for Population Health Sciences and Informatics, University of Edinburgh, Teviot Place, EdinburghEH8 9AG, UK,; 7 Department of Immunology, Genetics, and Pathology, Biomedical Center, SciLifeLab Uppsala, Uppsala University, Uppsala SE-75108, Sweden and; 8 Max-Planck-Institute for Molecular Genetics, Otto-Warburg-Laboratory, Berlin, Germany

## Abstract

Homozygous loss of function (HLOF) variants provide a valuable window on gene function in humans, as well as an inventory of the human genes that are not essential for survival and reproduction. All humans carry at least a few HLOF variants, but the exact number of inactivated genes that can be tolerated is currently unknown—as are the phenotypic effects of losing function for most human genes. Here, we make use of 1432 whole exome sequences from five European populations to expand the catalogue of known human HLOF mutations; after stringent filtering of variants in our dataset, we identify a total of 173 HLOF mutations, 76 (44%) of which have not been observed previously. We find that population isolates are particularly well suited to surveys of novel HLOF genes because individuals in such populations carry extensive runs of homozygosity, which we show are enriched for novel, rare HLOF variants. Further, we make use of extensive phenotypic data to show that most HLOFs, ascertained in population-based samples, appear to have little detectable effect on the phenotype. On the contrary, we document several genes directly implicated in disease that seem to tolerate HLOF variants. Overall HLOF genes are enriched for olfactory receptor function and are expressed in testes more often than expected, consistent with reduced purifying selection and incipient pseudogenisation.

## Introduction

The human genome contains more than 20 000 protein-coding genes, but it is currently unknown how many of these genes are essential for development, survival and reproduction, and how many genes are, to some degree, ‘dispensable’. All humans carry genetic variants predicted to cause loss of function (LOF) for a variety of protein-coding genes, i.e. they carry frameshift or premature stop codon mutations in the coding regions of genes, whole gene deletions, or splice site disruptions (1). In addition it has been estimated that each human genome carries around 20 of these LOF variants in the homozygous state (HLOF), resulting in a naturally occurring ‘knockout’ of the gene concerned (2). Compared with Mendelian disease variants, the phenotypic consequences of such HLOF variants may often be small, though HLOFs may have detectable effects upon more subtle phenotypes. Under certain scenarios, loss of gene function can have protective effects against disease, and such genes are of particular interest as novel therapeutic drug targets. For example, HLOF at *PCSK9* can lead to reduced levels of LDL cholesterol (3), HLOF at *SLC30A8* is protective for type 2 diabetes (4) and a homozygous partial deletion of *CCR5* protects against HIV infection (5,6).

Previous efforts have been made to catalogue HLOF variants in human populations (2,7), identifying a total of 253 and 169 homozygous loss of function genes within cosmopolitan individuals and individuals with consanguineous parents, respectively. Most HLOFs were present at very low frequencies, but the number of HLOFs per individual was higher in the study of offspring from consanguineous marriages and associated with high levels of homozygosity genome-wide (7). Only a modest overlap was evident between the genes identified in these two studies [only one-third of the HLOFs of (7) were also found by (2)], suggesting that many HLOFs are population-specific and that a wider variety may be discovered across diverse populations.

A recurring complication in the identification of HLOF variants is a high rate of false positives, caused by variants that are either sequencing errors or variants wrongly annotated as HLOFs. Sequencing errors are expected to occur relatively uniformly across the genome, but the proportion of true variation at highly constrained sites is expected to be much lower than at functionally neutral sites, i.e. a high proportion of sequencing errors can spuriously appear as HLOF variants (8), and further biases are introduced by the fact that HLOF mutations often occur at low allele frequencies (9,10). However, even if a HLOF variant is correctly called, some seemingly damaging mutations may have little effect on the function of the protein if they can be rescued by other, nearby variants, or if they occur in more ‘dispensable’ sections of a protein, such as those in rare isoforms or HLOF mutations at the end of an open reading frame. Hence, strenuous filtering and validation of putative loss of function variants is essential.

Population isolates provide natural laboratories for studying the roles of rare variants in complex phenotypes because the population's evolutionary history may often lead to relatively high frequencies of rare homozygous variants. On the one hand, population isolation leads to increased levels of background relatedness and inbreeding, which increases levels of homozygosity; in addition, genetic drift is stronger in small populations, which can lead to a higher frequency of mildly deleterious variants, such as LOF mutations, in small or bottlenecked populations (11). Indeed, a recent study found a significant enrichment of low frequency LOF variants in the Finnish population relative to other Europeans, and, by making use of phenotype data in 36 262 individuals, they could identify variants affecting quantitative traits (12).

In this study, we used exome sequencing in four European isolate populations spanning the continent and a collection of more cosmopolitan Scottish samples from the Generation Scotland: Scottish Family Health Study (GS:SFHS) (13). For the first time we are able to examine the frequencies of HLOF variants across isolate populations sampled from diverse sites across Europe and compare them to a national collection. Using exome data for a total of 1432 individuals, we identify 94 validated premature stop-gain and 79 frameshift variants; a total of 76 of which had not been observed in previous surveys (2,7), including the published variants of a study of Finnish and non-Finnish Europeans (12). We find a higher prevalence of HLOF variants in the isolates, and find that novel HLOFs are significantly enriched in these populations. We also exploit phenotypic data, to explore the effects of HLOF variants on hundreds of clinically relevant quantitative traits across many physiological areas. We find that HLOFs in our dataset generally have little effect on phenotype and appear to constitute largely neutral variation.

## Results

Across the 1432 exomes, 1 465 905 indels and single nucleotide polymorphisms (SNPs) passed the GATK default filters. These included predicted 1084 stop-gain and 1185 frameshift variants that were homozygous in at least one individual. However, the majority of these apparent HLOF variants were likely to be second-generation sequencing errors, rare errors in the reference genome or variants that do not disrupt gene function. Conservative filtering and additional conventional Sanger sequencing of variants led to the exclusion of most SNPs and indels from our analysis, reducing the number of homozygous stop-gains to 94 and the number of frameshifts to 79. In particular, Sanger sequencing revealed the importance of sequence coverage in variant calling accuracy (Supplementary Material, Fig. S1), and we conservatively set the minimum coverage for novel variants, not previously observed (2,7) to 20×. In addition, we removed three variants that were fixed or nearly fixed in all populations (only one heterozygote was observed for one of the three variants, all other sites were non-reference), assuming that these are either errors or rare variants in the reference sequence (at hg19 positions chrX:82764038; chrX:104464281; chr11:104763117). The number of variants that were removed by each of our filters is shown in Table 1.

**Table 1.** Filtering putative HLOF variants

Filter	Stop gain mutations	Frameshift mutations
Total number of variants before filtering	1084	1185
Relative_position < 0.9	883	973
Duke (mapability)	960	1027
DAC excluded regions (mapability)	1083	1175
CRg (mapability)	893	917
Min. 200	945	942
SureSelect & TrueSeq	794	829
Ancestral allele	1032	NA
MNP/frameshift nearby	982	817
Hardy–Weinberg	323	373
All filters applied, Sanger Sequencing	94	79

The number of putative HLOF variants remaining after each mutational filter had been applied individually. Filters have the following meaning: ‘Relative_position < 0.9’ indicates that variants are limited to the first 90% of all splice variants of a gene. ‘Duke’, ‘DAC’ and ‘CRg’ are mapability scores of UCSC. ‘Min.200’ indicates that at least 200 individuals were sampled for each variant. ‘SureSelect & TrueSeq’ is the intersection of the two exome sequencing kits. ‘Ancestral allele’ indicates whether the LOF variant is found in other primates. The ancestral allele filter was applied to frameshift mutations only after all other filters had been applied, leading to the exclusion of seven variants. ‘MNP/frameshift nearby’ indicates whether a restoring variant was found in proximity of the focal variant. ‘Hardy–Weinberg’ indicates whether the variant passed our Hardy–Weinberg filter.

Thirty-three variants were selected for Sanger sequencing based on their complete novelty or because they were present in dbSNP, but without validation status. Interestingly, among the 13 HLOF variants that were confirmed by Sanger sequencing as true positives, none were specific to the cosmopolitan GS:SFHS population, whereas 9 were found exclusively in the isolates (CROATIA-Vis, CROATIA-Korčula, NSPHS, ORCADES), at very low frequencies (Table 2). This is despite a lower number of individuals that were sequenced in isolate populations—588 in isolates versus 844 individuals in GS:SFHS—suggesting an enrichment of rare, novel HLOFs in isolate populations (Fisher's exact test, *P* < 0.01). Note that the variant at chr6:31106500 had a dbSNP validation record but was included in the Sanger-sequencing as a control; this variant was observed in four populations. Given that the Sanger sequenced variants were *a priori* expected to be enriched for false positives, their low validation rate is not too surprising. Among the 20 variants that failed validation by Sanger sequencing, 12 were completely absent (9 erroneously called stop-gains and 3 frameshifts); a further 3 frameshift variants were in fact heterozygous, and 2 deletions turned out to remove a multiple of 3 base pairs; 3 sequences were not clean or gave ambiguous sequencing results and were conservatively excluded. Other than low sequence coverage of these 20 variants, however, there were no indications as to why high throughput sequencing had given erroneous results.

**Table 2.** Sanger-sequencing confirmed HLOFs

Position	Gene	Number of homozygotes	Population
chr1:55076137	*FAM151A*	1	NSPHS
chr4:113539281	*C4orf21*	1	CROATIA-Korčula
chr5:96222446	*ERAP2*	1	CROATIA-Vis
chr6:28358464	*ZSCAN12*	1	CROATIA-Vis
chr6:31106500	*PSORS1C1*	26	CROATIA-Vis, ORCADES, GS:SFHS, NSPHS
chr12:70088219	*BEST3*	2	CROATIA-Vis, ORCADES
chr14:57672624	*AL391152.1*	2	NSPHS, GS:SFHS
chr15:44091290	*SERINC4*	1	CROATIA-Vis
chr17:46882286	*TTLL6*	2	CROATIA-Vis, GS:SFHS
chr17:47921435	*TAC4*	1	CROATIA-Vis
chr19:36230499	*IGFLR1*	1	ORCADES
chr19:51729103	*CD33*	3	NSPHS, GS:SFHS
chrX:50659021	*BMP15*	1	ORCADES

The number of individuals which were homozygous for a novel, Sanger-sequencing confirmed variant, and the population where the variant was found.

Individuals from population isolates tend to carry longer runs of homozygosity (ROHs) compared with more cosmopolitan individuals (14). Rare HLOFs are expected to be enriched within ROHs because they represent regions that are identical-by-descent, that is, inherited from the same common ancestor (7); thus, rare variants, which would otherwise not be found in the homozygous state, are brought together (15).

**Figure 1.**
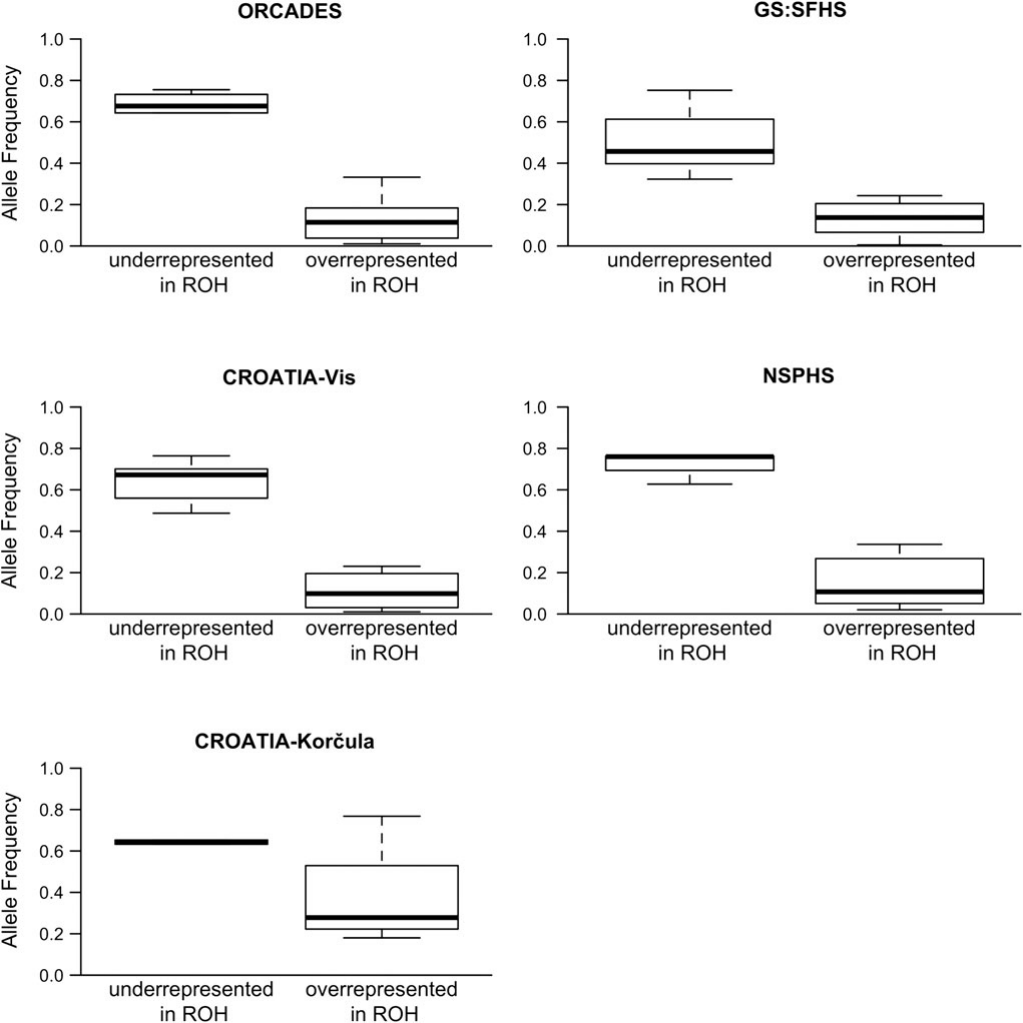
Rare HLOFs are found within ROHs. Allele frequencies of HLOFs that are biased towards being inside or outside runs of homozygosity (ROHs) in the five populations studied (using a binomial test with *P* < 0.1). For GS:SFHS, CROATIA-Vis, ORCADES and NSPHS, the allele frequencies of variants that were enriched in ROHs were significantly lower compared with variants that were found in the autozygome (Wilcoxon test; *P* < 0.05). In CROATIA-Korčula, only two variants were underrepresented in ROHs, and the Wilcoxon test was not significant.

To test this, we assessed, for each HLOF and carrier, whether a variant was found within or outwith a run of homozygosity (ROH). We then calculated the overall frequency of HLOFs which fell within ROHs; this value differed among populations (3% of HLOFs in GS:SFHS were found within ROHs, whereas it was 10% in CROATIA-Korčula; 8% in NSPHS; 7% in CROATIA-Vis; 6% in ORCADES), reflecting the different overall proportions of the genome that are found within ROHs in isolate versus our cosmopolitan sample (14) (Fisher's exact test comparing the number of HLOFs inside and outside ROHs in GS:SFHS versus each isolate population: *P* < 0.01 in each comparison). Singleton HLOFs that were observed only once across all populations were found more often within ROHs (15 out of 48 HLOFs = 31%)—and all seven novel singletons identified in this study fell within ROHs. Importantly, HLOF variants that were biased towards being inside ROHs had lower allele frequencies compared with variants that were mostly found outside ROHs (Fig. 1), suggesting that rare HLOFs can be found more easily in individuals with increased homozygosity.

**Figure 2.**
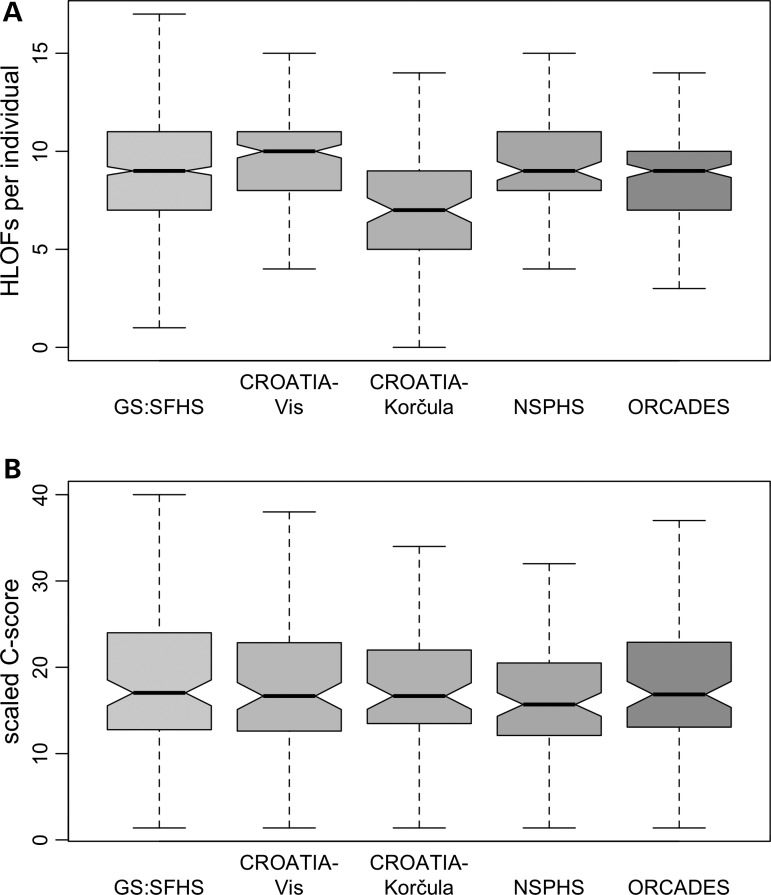
Number of HLOFs per individual and predicted deleteriousness. Boxplot of the number of HLOFs carried by each individual in GS:SPHS, CROATIA-Vis, CROATIA-Korčula, ORCADES and NSPHS (**A**) and the C-scores associated with HLOF variants in the five populations (**B**).

Table 3 shows the number of HLOF variants found in each of the five populations. Notably, the yield of HLOF variants, i.e. the number of observed HLOFs per number of individuals sampled, was much higher for the isolates compared to GS:SFHS: 0.52 to 0.93 compared with 0.16 per individual, respectively. However, the average burden (i.e. number of HLOFs) did not differ between GS:SFHS and the isolates: each individual carried a median number of nine HLOFs in GS:SFHS, NSPHS and ORCADES; the median number of HLOFs was 10 in CROATIA-Vis and seven in CROATIA-Korčula (Fig. 2). Note that the lower value in CROATIA-Korčula was probably caused by the lower sequence coverage for samples from this population, resulting in more sites being excluded from the analysis. Hence, even though individuals from isolate populations did not carry a higher absolute number of HLOFs, the mutational spectrum that was captured differed, resulting in an enrichment of rare (and thus potentially more likely to be functional) HLOFs in isolates.

**Table 3.** Summary of the numbers of HLOFs found

Population	*N*	*N*(HLOFs)	Yield	Private LOFs	Private HLOFs
GS: SFHS	844	137	0.16	1	26
CROATIA-Vis	193	104	0.54	2	8
ORCADES	197	103	0.52	3	13
NSPHS	98	91	0.93	0	6
CROATIA-Korčula	100	74	0.74	1	4

The sample size, *N*, and the number of HLOF mutations that were found in each population, N(HLOFs); the average yield per individual (*N*(HLOFs)/*N*); the extent to which the mutations are shared across populations. Private LOFs are seen only in one population, including as heterozygotes; private HLOFs are shared as heterozygotes across populations.

To assess the predicted consequences of our HLOF variants, we examined their C-scores (16), which indicate the ‘deleteriousness’ of a given (multi)nucleotide change. HLOF variants were found to possess C-scores between 1.4 and 44.0, with an overall median score of 18 (Fig. 2). In comparison, the top 1% of deleterious variants in the genome are assigned a score of ≥20; the predicted median C-score of nonsense variants within known disease genes (defined as genes that harbour at least five known pathogenic mutations) is 37 (16,17). In our dataset, the maximum C-score of 44 was found in a variant within the *WDR87* gene on chromosome 19 (rs151219712), which was homozygous in a single individual from GS:SFHS (Supplementary Material, Table S1). The median C-score of our novel, Sanger sequencing-confirmed, variants was 20.0; this is not significantly different from that of the remaining, previously known, variants, which had a median score of 18.0 (Wilcoxon test: *W* = 1041.5; N.S.). However, there was a negative correlation between the C-score and the number of individuals carrying a HLOF variant (Spearman's rho = −0.30; *P* < 0.05), consistent with the extent of purifying selection on a variant being reflected, to some extent, by the number of times an HLOF is observed.

Gene Ontology analysis using GOrilla (http://cbl-gorilla.cs.technion.ac.il/ accessed September 2014) (18) revealed an excess of transmembrane signalling receptor genes amongst HLOF genes, including olfactory receptor genes (Table 4). Indeed, olfactory receptor genes have previously been shown to be over-represented among HLOF genes (2), and segregating polymorphisms of functional and non-functional copies of olfactory genes are common (19). Although no other GO category was over-represented among the HLOF genes discovered here, a large number of HLOF genes were expressed in testis: across 16 tissues sampled, expression was highest in testis for 28.7% of HLOF genes, i.e. for 41 out of the 143 HLOF genes that were present in the EBI expression atlas (http://www.ebi.ac.uk/gxa/ accessed September 2014) (20,21). The proportion of testis-expressed genes amongst all genes sampled was also higher for the HLOF genes (119 out of 143 genes = 83%) compared with the overall expression profile of human protein-coding genes (77% of all human transcripts detected by (22) were found in testis, with 10% being enriched in testis). This apparent loss of function at testis-expressed genes is consistent with a recent study (23), which showed that male-specific genes in humans carried a relatively high load of deleterious polymorphisms, possibly due to reduced selection on these variants in females who do not express these genes.

**Table 4.** Gene Ontology

GO term	Description	FDR adjusted *q*-value	Enrichment (*N*, *B*, *n*, *b*)
GO:0004984	Olfactory receptor activity	3.35E-11	7.65 (17 424,362,151,24)
GO:0004930	G-protein coupled receptor activity	7.86E-06	3.81 (17 424,788,151,26)
GO:0004888	Transmembrane signalling receptor activity	8.29E-04	2.80 (17 424,1154,151,28)
GO:0004872	Receptor activity	8.60E-04	2.52 (17 424,1464,151,32)
GO:0038023	Signalling receptor activity	2.50E-03	2.58 (17 424,1253,151,28)
GO:0004871	Signal transducer activity	9.62E-02	2.09 (17 424,1548,151,28)
GO:0060089	Molecular transducer activity	8.25E-02	2.09 (17 424,1548,151,28)

GO analysis of genes containing HLOF variants, using, as a background set, all genes captured by the intersection of the SureSelect and TruSeq exome sequencing kits.

Enrichment = (*b*/*n*)/(*B*/*N*); *N* = Number of genes; *B* = Number of genes associated with a GO term; *n*= number of genes in the target set; *b* = number of genes in the intersection.

Only nine HLOF genes discovered here are annotated as disease-associated in the scientific literature, with an entry in the OMIM database (http://www.omim.org/ accessed November 2014), indicating the relatively low impact of HLOFs found in healthy individuals. Further, it should be noted that most OMIM genes in our dataset were associated with risk phenotypes, rather than Mendelian diseases: *BTNL2*, *CARD14*, *TLR5* and *ARMS2* were associated with susceptibility to sarcoidosis, psoriasis or legionnaire disease and late onset macular degeneration, respectively; *RNF212* was associated with a non-risk phenotype, variation in recombination rate (Supplementary Material, Table S1).

A wide range of phenotypic measurements were available for individuals from the five populations studied (Supplementary Material, Table S2). However, there was no evidence for any variant possessing a measurable effect, including four variants in known disease genes:

The Macrophage Scavenger receptor 1 (MSR1, also known as SRA) is an LDL receptor, and is implicated in the pathologic deposition of cholesterol in arterial walls (24,25). A single individual (a 68 year-old female from CROATIA-Vis with a body mass index of 24.5) carried a HLOF in MSR1; this variant had not previously observed as a homozygote (2,7,12), and it was associated with a high C-score (38.0). However, the individual showed no suspicious phenotype, and her serum lipid levels were normal (total cholesterol 5.7 mmol/l; HDL 1.92 mmol/l; LDL 1.48 mmol/l), suggesting that losing function at the *MSR1* gene does not have major effects on blood lipid levels.

Bone morphogenetic protein 15 (*BMP15*) is an X-linked gene, which is exclusively expressed in ovaries, in the oocytes of late primary follicles (26), and it is associated with infertility and ovarian dysgenesis (OD), even in heterozygotes (27–29). In our dataset, we found a rare LOF variant in *BMP15* in ORCADES: in the exome sequence dataset, a single male carried a (hemizygous) LOF frameshift variant in the mature protein-coding region of *BMP15*, upstream of the missense mutation described previously (29). The same variant was also found in the heterozygous state in three ORCADES females, but in no other population. We Sanger sequenced these 4 individuals, as well as 33 additional Orcadians from the ORCADES study, all of whom were first, second or third degree relatives of the 4 known carriers. Supplementary Material, Table S3 shows their genotypes and the number of offspring: we never observed the frameshift variant in the homozygous state in females; and of the five heterozygous females, two had children. Further, the variant was present on the single X chromosome in nine males (whose mothers must have carried the variant on at least one X), so we can conclude that the frameshift variant does not lead to OD in heterozygotes.

Among the HLOF variants in genes with OMIM entries, two variants might be rescued by alternative transcripts and reading frames. *PDE6G*, the first of these genes, encodes one of the three subunits of Cyclic GMP-phosphodiesterase (PDE), and incorrect splicing of the gene is reported to lead to early-onset retinitis pigmentosa in a family-based mapping study (30). In our dataset, a HLOF was observed in six individuals, but the four carriers who were scored for eye phenotypes (from CROATIA-Vis, CROATIA-Korčula and ORCADES) did not show any abnormalities. However, the frameshift variant was detected in only one of the five alternative transcripts for this gene, and function may be restored by the presence of alternative transcripts for which the variant is intronic and in the 5′ UTR respectively. Similarly, variants in *SERAC1* are thought to cause a severe Mendelian phenotype (MEGDEL syndrome), which includes encephalopathy, dystonia and deafness (31). We find a HLOF in a single individual from CROATIA-Vis—a healthy 33 year-old male who showed no unusual phenotype. However, *SERAC1* has several transcripts in two different reading frames, and the stop-gain variant occurs a reading frame, which is expected to lead to nonsense-mediated decay (in transcripts ENST00000607071, ENST00000607742, ENST00000606965). The same variant is merely a missense variant on exon 4 of the transcript (ENST00000367104), i.e. outside the lipase/esterase domain, which is implicated in causing MEGDEL syndrome when disrupted (31).

In all of the five populations, the number of HLOFs for a given individual (which ranged from 0 to 18) was not a good predictor of the observed number of outlier traits per individual, i.e. the number of trait values in the top or bottom 1% of the population (which ranged from zero to 211); this result remained unchanged also if only strongly deleterious HLOFs (C-score >25) were included in the analysis, or if olfactory genes were excluded (Supplementary Material, Table S4). If the majority of genes containing HLOF variants do not have measureable phenotypic effects, this could be due to a higher than usual number of paralogues for these genes, i.e. genes belonging to the same gene family could act as a buffer on the effect. This, however, does not seem to be the explanation: when olfactory genes were excluded, the number of paralogues for HLOF-containing genes was actually lower compared with protein-coding genes in the genome as a whole (Wilcoxon test, 70% identity cut-off for paralogues: *P* < 0.05; Wilcoxon test, 80% identity cut-off for paralogues: *P* < 0.01). Further, the number of one-to-one orthologues with mouse and chimp did not differ between the set of HLOF genes and all human protein-coding genes on Ensemble (Chi-square test with Yates's correction: N.S. for both comparisons).

## Discussion

Using exome sequences from 1432 individuals, we extend the known repertoire of human genes that are dispensable, i.e. genes that can carry homozygous loss of function variants in healthy individuals. The vast majority of these genes do not have strong phenotypic effects, i.e. they seem to be truly dispensable. Further, all but seven HLOF variants are shared across the five European populations as heterozygotes (Table 3), i.e. most HLOFs are relatively old and hence unlikely to be strongly deleterious. In line with this, the predicted deleteriousness of HLOF variants is, on average, lower than that of predicted disease variants. However, we do find the isolate populations to be enriched for homozygous LOF variants of low frequency because rarer variants are relatively more likely to be brought into the homozygous state within the long ROHs present in these populations. Accordingly, population isolates are a good place to look for novel HLOF variants. Of course, it would be advantageous to extend the search for HLOF genes beyond those of European heritage populations, especially those with high levels of homozygosity: extended ROHs have been found in other worldwide populations, such as Native American and Oceanian populations (32,33); homozygosity in these populations is presumably caused by bottlenecks and haplotypes randomly drifting to a high frequency. Even longer ROHs tend to be found in the Near and Middle East, which has a history of recent consanguinity, and has already been used to study HLOFs (7). Intriguingly, the distribution of ROHs among populations is non-uniform (32): first, this might simply be due to chance, i.e. rare recombination events can break up different associations in different populations; in addition, regions of high levels of homozygosity often overlap with loci of recent positive selection—which are often population-specific and include loci such as the human skin pigmentation gene, *KITLG* (32). Presumably, rare HLOF variants are enriched in segments of recent positive selection, for example, due to genetic hitch-hiking (34). The best way to find these variants is by sequencing more individuals from a broader geographical spectrum, focussing on isolated populations that are geographically distant from Africa and/or populations that have experienced recent inbreeding.

Overall, we find a similar total number of HLOF variants as a study of consanguineous individuals (7), but many fewer than a large study of cosmopolitan individuals (2). This may be partly due to a slightly different set of filters applied; some of our filtering was very conservative, and this might have led to a relatively higher rate of exclusion of true variants. For example, neither of the two other studies removed variants that showed an excess of homozygous LOF variants in the population (the Hardy–Weinberg filter), but we show that this filter is indeed a good indicator of an erroneous SNP call. Further, we excluded all HLOF variants that occurred in the last 10% of any transcript of a gene, whereas the 1KG study (2) removed variants if they were found in the last 5% of the longest transcript for a particular gene. We also applied a mapping-based filter, i.e. the ‘uniqueness score’ of the UCSC genome browser, which had not been used before; further, the prior cosmopolitan survey also included splice sites and deletions in their set of HLOFs (2), which increases the overall number of HLOFs they report. Indeed, 29 HLOF variants of the consanguineous dataset (7) were removed by our approach, and 203 variants in the 1KG dataset (2) were removed from our homozygous set of LOFs; note that this included variants that were seen as heterozygotes in the 1KG data (2).

Despite differences in approach, it becomes clear that HLOF variants are relatively common amongst healthy individuals, with each individual carrying about 10–20 HLOFs, and many seemingly deleterious mutations can be tolerated. Even though these variants may not have any measurable effect, cataloguing them will help identifying rare mutations, which do cause severe phenotypes and diseases, by acting as negative controls. Of course, the effect of losing function at a given gene may also depend on the genomic background, i.e. other variants present in the same genome, and therefore dispensability may be a complex phenomenon.

In addition, disruptive mutations affecting the same gene may not always have the same phenotypic consequences, as outlined in our study of *BMP15*: In a previous study (29), a rare, heterozygous, missense mutation was shown to lead to OD due to abnormal processing of the precursor BMP15 protein, which resulted in precursor dimers and/or precursor-mature peptide heterodimers. Importantly, the missense mutation reported in (29) was associated with reduced granulosa cell growth even in the presence of wild-type BMP15, i.e. in heterozygous assays. However, Orcadian females, who are heterozygous for the novel mutation described here, are fertile—even though their frameshift variant is upstream of the previously described missense mutation. There are two possible explanations to this. First, in contrast to the missense mutation, the frameshift variant will lead to a completely altered protein sequence and structure. Accordingly, oocytes will be completely free of abnormal BMP15 hetero-dimers in heterozygotes, of the kind that had been observed before (29), i.e. losing one copy of *BMP15* might be less deleterious than having one copy that is compromised in its function. Alternatively, given that genes in the vicinity *BMP15* are subject to X-inactivation (35) and silencing of one X often occurs in a block-wise and skewed manner (36), it is possible that the copy of *BMP15* containing the frameshift variant became preferentially silenced in Orcadian females, whereas the missense mutation was expressed to a greater extent in the heterozygous females who developed OD. Accordingly, the genomic context and exact type of mutation may ultimately affect the phenotypic outcome for loss of function variants.

Our results suggest that buffering by paralogues genes cannot explain the presence of the HLOF variants because we actually observe a dearth of paralogues for HLOF genes. Partly, this may be explained by our mutational filter, which removed variants in non-unique genomic regions. However, it remains possible that HLOF genes are dispensable for other reasons, such as buffering by unrelated genes of similar function, or that function is rescued by alternative transcripts of the same gene; note that only 35 HLOF variants were present in all reported isoforms of a given gene, and 48 variants were observed in only one of two or more alternative transcript (Supplementary Material, Table S1). Further, phenotypic effects may not be picked up by our broad survey or are too subtle to be detectable when there are typically only one or two carriers: While it is relatively straightforward to show that a given HLOF does not inevitably cause a severe disease, it requires a large sample sizes and replicated studies to demonstrate variant-trait associations for quantitative traits. Accordingly, with hundreds of phenotypic traits measured for GS:SFHS and the isolate populations, we were able to search for phenotypic outliers; however, given the often small number of individuals carrying a given HLOF, we lacked statistical power to find genotype-phenotype associations in our dataset, which is a general caveat when studying rare variants with small effects.

This project is part of a current effort to catalogue and understand the impact of naturally occurring loss of function mutations in humans. Another possible field of research—loss of function at regulatory elements—has so far been neglected in population surveys, even though the effects of variants at non-coding sequence may potentially be just as severe as those in translated regions. This is certainly an area that will become more accessible with whole genome sequencing becoming cheaper—though the associated computational challenges will be even greater.

## Materials and Methods

We used whole exome sequences from GS:SFHS (13) and four isolate populations: two Croatian Islands (CROATIA-Vis and CROATIA-Korčula) (37,38), the Northern Swedish Population Health Study (NSPHS) recruited in the northernmost two counties of Sweden (39) and the Orkney Complex Disease Study (ORCADES), sampled in the Orkney Isles (Orcadians) (37). Originally, these individuals had been recruited for population-based studies of complex traits, i.e. a range of phenotypic data had been collected, including anthropometry, lipids, glycaemic traits, body composition, blood biochemistry, glycomics, cognitive function, etc. (Supplementary Material, Table S2). Notably, these individuals were generally healthy (from population-based sampling), except for some in the GS:SFHS cohort, which included obese individuals, as well as individuals with major depression. The Generation Scotland cohort 1 (GS:SFHS 1) contains 432 samples that were sequenced at the GenePool facility (University of Edinburgh) using the Illumina TruSeq capture kit (Illumina, CA, USA) with a mean coverage per sample of ∼38×. Generation Scotland cohort 2 (GS:SFHS 2) contains 428 samples that were sequenced at the Wellcome Trust Sanger Institute, as part of the UK10K project, using the Agilent SureSelect capture kit v3 (Agilent Technologies, CA, USA), with a mean coverage of ∼86×. A total of 588 individuals were sequenced from isolate populations: 193 from CROATIA-Vis, 107 from ORCADES, and 98 from NSPHS were sequenced at the Wellcome Trust Sanger Institute using the Agilent SureSelect capture kit v3 with a mean coverage of ∼59×. Ninety ORCADES samples and 100 from CROATIA-Korčula were sequenced at Source Biosciences, Nottingham, using the Agilent SureSelect v2 kit with a mean coverage of 30×.

Reads were aligned to the human reference genome GRCh37 using bwa (version 0.62 for CROATIA-Korčula and version 0.59 for all other samples) (40). For each aligned sample, duplicate reads were marked with Picard (version 1.79), and Samtools 0.1.16 (41) was used at various points along the analysis pipeline, e.g. the merging or indexing of bam files. Realignment around insertions/deletions (indels), base quality score recalibration and variant discovery were performed using the Genome Analysis Tool Kit (GATK) 2.7.2, according to GATK best practice recommendations for exome sequence analysis (42,43). Variant discovery was carried out for the target intervals covered by either TruSeq or SureSelect, with an additional 50 bp of padding around the target regions. SNP and indel calling was performed with UnifiedGenotyper, using reduced alignments and downsampling (to 250) across all 1432 samples simultaneously. Variant recalibration was performed with GATK version 2.8.1 and dbSNP version 137 was used throughout this pipeline.

Bam and vcf files were submitted to the European Nucleotide Archive (ENA) at the European Bioinformatics Institute (http://www.ebi.ac.uk/), with accession numbers XXX (to be added upon publication).

We extracted all sequence variants (SNPs or indels) which passed the GATK recalibration, showed a homozygous non-reference allele in at least one individual and were predicted to introduce premature stop-gain or a frameshift into the coding region, as determined by the variant effect predictor (44), using Ensembl 75 gene models. We did not consider splice disruption mutations since they would have required validation using expression data in multiple tissues. Multi-allelic variants were separated out into their respective variants using bcftools, and the remaining homozygous stop-gain and frameshift variants constituted our pool of putative HLOF mutations.

Next, variants were subjected to a range of filters, in order to remove false positives due to sequencing and variant calling errors, annotation errors, as well as variants that are unlikely to cause a true loss of function to the protein (Table 1). In particular, because of the expected high rate of false positives in the initial dataset, we set our filters to be very conservative, and describe these in the following.

Protein sequences for transcripts affected by putative stop-gain or frameshift mutations were downloaded from Ensembl biomart (http://www.ensembl.org/biomart, accessed June 2014), and the relative position of the variants assessed. Variants often clustered near the end of the end and beginning of a transcript (Supplementary Material, Fig. S2), reflecting reduced functional consequences for variants located in the extreme and of a reading frame, alternative start codons (2), or a lower sequence coverage near the start codon. We only kept variants if they fell into the first 90% of all transcripts annotated for a particular gene; notably, the excess of variants near the start codon were removed by other filters that we applied.

In order to avoid spurious calling of SNPs due to mapping of reads from paralogous genomic regions, we downloaded UCSC tracks of Crg (36mer) and Duke (35mer) Alignability scores, as well as DAC blacklisted regions; SNPs and indels that fell into non-unique regions of the genome (with Alignability scores <1.0) were excluded from further analyses, as were variants within in UCSC blacklisted regions.

To remove sites that are potentially difficult to sequence and hence more prone to sequencing or alignment errors, we restricted our set of HLOF variants to sites that were called in at least 200 individuals, and, to make a comparison possible across populations, we only considered sites that were captured by both exome capture techniques (TrueSeq and SureSelect), plus 50 bp of padding around the target region.

By the intersection, the target region was reduced from a total of 51.5 MB (for SureSelect) and 62.1 MB (TruSeq) to 33.4 MB.

We excluded variants that were also found in the genome assemblies of primate outgroup species (chimp, gorilla, orang-utan). These variants are potentially mis-annotated in the human genome (e.g. due to sequencing errors), or the variant that we detect might be an ancient polymorphic stop loss mutation in the primate lineage. For the primate comparison, vertebrate multiZ alignments were downloaded from the UCSC genome browser, and the human variants were compared to those in the other three primate species. To allow for alignment ambiguity in the case of frameshift variants, 5 bp upstream and downstream of an indel were extracted and compared. A frameshift was filtered out if (1) at least two primates had aligning sequence present in the region, (2) all species, which showed an alignment, carried the non-reference variant and (3) the position, length and type (insertion or deletion) of the primate variant matched the one seen in humans (according to (2)). Stop-gain variants were filtered out whenever one or more of the primate species carried the human non-reference allele.

Multinucleotide polymorphisms (MNPs), i.e. variants that consist of two or more adjacent SNPs, were filtered out if a stop-gain variant was found within the same codon as another SNP. For frameshift variants, the corresponding filter was slightly more sophisticated. In the simplest case, a transcript contained only a single frameshift variant, which was then kept. Frameshifts were also kept for further analyses if (a) a transcript contained two or more homozygous frameshift variants that were separated by 10 or more amino acids in the translated protein or (b) nearby frameshift variants (<10 amino-acids apart) did not result in a restoration of the reading frame, i.e. the sum of bases inserted or deleted was not a multiple of three. In contrast, frameshift variants were filtered out (i) if two nearby variants resulted in the restoration of the reading frame or (ii) whenever three or more frameshifts occurred within close vicinity of each other because insertions or deletions resulting in triplets were possible for these variants.

The high false positive rates due to sequencing errors among LOF mutations can, at least partially, be corrected by considering the overall allele frequencies and contrast these with the frequencies of homozygous versus heterozygous individuals within a population, i.e. testing for Hardy–Weinberg equilibrium (HWE). Accordingly, we filtered out any putative LOF variants that were found in more homozygous individuals than expected under HWE in any of the five population samples considered separately (with a *P*-value <0.01). This approach is based on the assumption that loss of function mutations are not subject to positive selection, i.e. they are either removed by selection (leading to a reduction in the number of homozygotes) or effectively neutral. An excess of homozygotes can also be observed when there is population substructure; however, as a control, we show that the variants confirmed previously (2,7) had fewer deviations from HWE than our unfiltered, raw variants (Supplementary Material, Fig. S3), which is why we conservatively applied the Hardy–Weinberg filter. For the allele frequency calculations, we included all individuals with a coverage of at least 5×; however, to be kept in the analysis, a variant had to be observed in a homozygous state in at least one individual with a coverage of 20×.

Last, to validate our filtered set of HLOF variants, we Sanger-sequenced any novel variants that we detected in our samples; these included 33 variants which were neither found in the set of HLOF variants of (2) nor (7), and had no validation record in dbSNP. Note that these variants constitute the set of variants that is, *a priori*, most biased towards containing false positives. Primers were designed within ∼400 bp surrounding each loss of function variant using Primer3 (45,46). Sequences were amplified by PCR and bidirectional sequencing performed using N13 primers and a Big Dye Terminator Cycler Sequencing kit v1.1 (Applied Bio-systems, Warrington, UK). Results were compared with the reference sequence (NM_000545.3) Sequencher v4.8 (Gene Codes Corporation, Ann Arbor, MI, USA) and large heterozygous deletions were analysed using Poly Peak Parser (http://spark.rstudio.com/yostlab/PolyPeakParser/ accessed September 2014). A hundred and five other homozygous null variants identified by exome sequencing were validated and confirmed by comparison to Illumina exome array and/or Illumina Omni Express genotyping data in Generation Scotland, CROATIA-Korčula and ORCADES.

Since the Sanger sequencing revealed the importance of sequence coverage in calling homozygotes correctly in the exome dataset (see Results section), we decided to only retain, in our final set of LOF variants, homozygous mutations that were (a) either Sanger confirmed (13 variants) or (b) observed and validated by previous LOF studies (2,7) (111 variants) or (c) variants that had a sequence coverage of 20 in at least one homozygous individual (49 variants). Note that none of our 62 novel variants were observed in the recent Finnish study (12). Except where stated otherwise, our inter-population comparisons, however, were performed setting the minimum coverage of these variants to 5, given that the mean sequence depth was only 19 in CROATIA-Korčula.

GO term enrichment analysis was performed using GOrilla (18) with 17 424 genes captured by the exome sequencing as the background set.

Runs of homozygosity (ROHs) were called using array data that had already been subjected to quality control. Genotyping was performed using the Illumina HAP300, Omni1 and OmniX arrays. Individuals with a call rate less than 98% were excluded, as were SNPs with a call rates less than 98%, minor allele frequencies of less than 5%, and SNPs failing a test of HWE (*P* < 10^−6^). Autosomal ROHs exceeding 1.5 Mb in length were called using PLINK, with the following settings: --homozyg-window-snp 50 --homozyg-snp 50 --homozyg-kb 1500 --homozyg-gap 1000 --homozyg-density 50 --homozyg-window-missing 5 --homozyg-window-het 1. We extracted HLOFs that were biased towards being found either within or outside ROHs respectively; for this means, we used a binomial test with an expected frequency equal to the overall frequency of HLOFs within ROHs (7). This frequency was calculated separately for each population. Next, we compared the allele frequencies of the two classes of HLOFs, i.e. HLOFs biased towards being found mostly within ROHs (Binomial test: *P* < 0.1) and those found mostly outside the autozygome (Binomial test: *P* < 0.1).

A number of the populations cohorts have been extensively studied, with a range of clinically relevant quantitative traits measured; to find possible traits associations, we used a combined dataset, which included all individuals whose exomes had been sequenced, as well as individuals who had been genotyped on Illumina HumanHap300, CNV370, OmniExpress or Omni1 arrays. The number of traits which we used for analysis were 867, 455, 194, 444 and 82 in a total of 2426 Orcadians, 1145 CROATIA-Vis islanders, 971 CROATIA-Korčula islanders, 1032 Swedes and 853 individuals of GS:SFHS respectively (Supplementary Material, Table S2). However, with very small sample sizes (number of carriers) for most HLOFs, there was limited power to detect any phenotypic associations; further, if each variant was to be tested independently, the number of potential tests performed would have been very large (867 traits tested for association with 173 variants in ORCADES), leading to spurious associations. Hence, our main strategy was to relate the overall mutational burden, i.e. the number of HLOFs carried by a given individual, to the number of extreme phenotypic values observed, i.e. the number of traits that fell into the bottom or top 1% quantile of the trait distribution for a given individual in a given population. Using R, we constructed a linear model, using the number of extreme trait values as a response variable, and, as predictor variables, the number of HLOFs, as well as the number of traits measured for an individual (accounting for the fact that not all individuals were measured for all traits). We performed the analysis separately for each population, and also repeated it, removing (1) all olfactory genes or (2) all variants with a C-score less than 25.

## Supplementary Material

Supplementary Material is available at *HMG* online.

## Funding

GS:SFHS is funded by the Scottish Executive Health Department, Chief Scientist Office, grant number CZD/16/6. SNP genotyping was performed at the Wellcome Trust Clinical Research Facility in Edinburgh and was funded by the Medical Research Council UK. The NSPHS study was funded by the Swedish Medical Research Council (project numbers: K2007-66X-20270-01-3 and 2011-2354) and the Foundation for Strategic Research (SSF). NSPHS as part of EUROSPAN (European Special Populations Research Network) was also supported by European Commission FP6 STRP grant number 01947 (LSHG-CT-2006-01947). This work has also been supported by the Swedish Society for Medical Research (SSMF). Illumina genotyping analyses was performed by the SNP & SEQ Technology Platform in Uppsala, which is supported by Uppsala University, Uppsala University Hospital, Science for Life Laboratory (SciLifeLab)—Uppsala and the Swedish Research Council (Contracts 80576801 and 70374401). The exome-sequencing data analyses of isolated populations used in this study were supported by the European Commission (FP7/2007-2013, under grant agreement number no. 262055 (ESGI), by two Transnational Access projects of the European Sequencing and Genotyping Infrastructure). This study makes use of data generated by the UK10K Consortium, derived from samples from UK10K_OBESITY_GS (EGAS00001000242). A full list of the investigators who contributed to the generation of the data is available from www.UK10K.org. Funding for UK10K was provided by the Wellcome Trust under award WT091310. ORCADES was supported by the Chief Scientist Office of the Scottish Government (CZB/4/276, CZB/4/710, CZB/4/438), the Royal Society (University Research Fellowship to J.F.W.); MRC core funding to the QTL in Health and Disease research program at the MRCHGU, IGMM, University of Edinburgh; Arthritis Research UK; the Volant Trust and the European Union framework program 6 EUROSPAN project (contract no. LSHG-CT-2006-018947). DNA extractions were performed at the Wellcome Trust Clinical Research Facility in Edinburgh. The CROATIA-Vis study was funded by grants from the Medical Research Council (UK) and Republic of Croatia Ministry of Science, Education and Sports research grants (216–1080315-0302). The SNP genotyping for the CROATIA-Vis cohort was performed in the core genotyping laboratory of the Wellcome Trust Clinical Research Facility at the Western General Hospital, Edinburgh, Scotland. The CROATIA-Korčula study was funded by grants from the Medical Research Council (UK), European Commission Framework 6 project EUROSPAN (contract no. LSHG-CT-2006-018947) and Republic of Croatia Ministry of Science, Education and Sports research grant (216-1080315-0302) and Croatian Science Council grant 8875. The SNP genotyping for the CROATIA- Korčula cohort was performed in Helmholtz Zentrum München, Neuherberg, Germany. Funding to pay the Open Access publication charges for this article was provided by the RCUK Open Access Publication Fund.

## Supplementary Material

Supplementary Data
