# Supplementary material for: Homozygous loss-of-function variants in European cosmopolitan and isolate populations
Source: Hum Mol Genet. 2015 Jul 14;24(19):5464–74. doi: 10.1093/hmg/ddv272 (PMC4572071; doi:10.1093/hmg/ddv272)
Supplement: Supplementary Data [file supp_24_19_5464__index.html]

Homozygous loss-of-function variants in European cosmopolitan and isolate populations — Homozygous loss-of-function variants in European cosmopolitan and isolate populations — Homozygous loss-of-function variants in European cosmopolitan and isolate populations — Supplementary Data 

# Homozygous loss-of-function variants in European cosmopolitan and isolate populations

## Supplementary Data

Supplementary Data

- Supplementary Data - Docx file
- Supplementary Table 1 - xlsx file
- Supplementary Table 2 - xlsx file
- Supplementary Table 3 - xlsx file
- Supplementary Table 4 - xlsx file
