# Supplementary material for: Homozygous loss-of-function variants in European cosmopolitan and isolate populations
Source: Hum Mol Genet. 2015 Jul 14;24(19):5464–74. doi: 10.1093/hmg/ddv272 (PMC4572071; doi:10.1093/hmg/ddv272)
Supplement: Supplementary Data [file supp_ddv272_ddv272supp.docx]

Supplementary Data

**Table S1. Summary of HLOFs identified in this study.**

The Table includes chromosomal positions in hg19; genes affected; C-scores of the HLOF variants; validation records in dbSNP and previous studies on HLOFs (1, 2); variants confirmed by Sanger sequencing; number of alternative homozygous heterozygous and reference alleles observed in the five populations studied; transcript abundance (FPKM) in sixteen tissues available at the EBI expression atlas at http://www.ebi.ac.uk/gxa/home (3, 4).

**Table S2. Phenotype traits scored in the five population samples.**

**Table S3. Genotype and fertility data on 37 Orcadians at the *BMP15* locus.**

**Table S4. Modelling the effect of HLOFs on phenotype.** Results of a linear model in R (“lm(formula = Number_outlier_traits ~ Number_HLOFs + Number_traits_scored”), using, as predictor variables, the number of HLOFs per individual (Number_HLOFs) and the number of traits measured (Number_traits_scored); the response variable was the number of observed outlier traits (Number_outlier_traits).

**Figure S1. Sanger validation.**

The proportion of putative HLOF variants that were successfully validated using Sanger sequencing, plotted against the minimum coverage of those variants in the exome dataset.


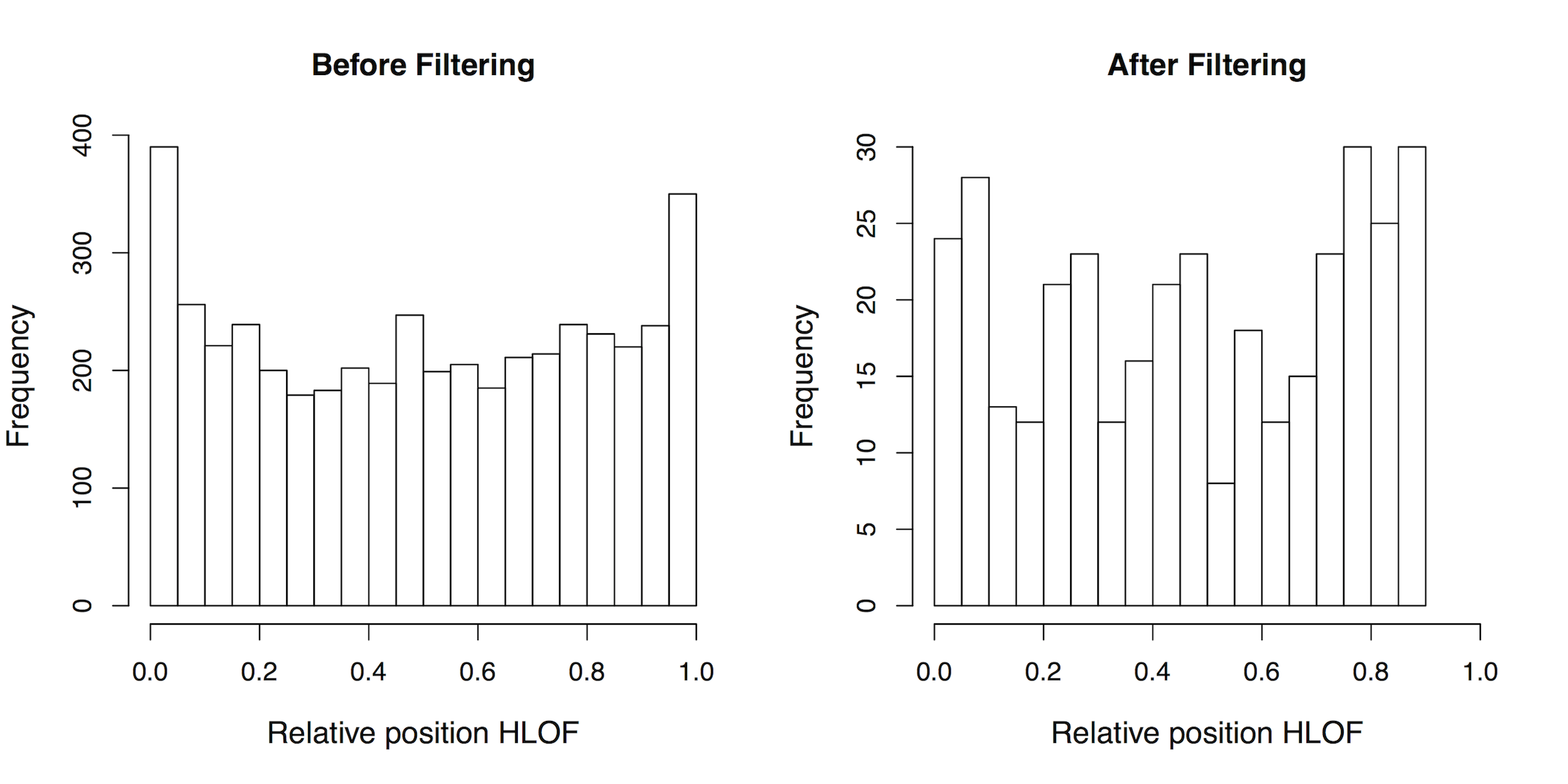


**Figure S2. Relative position within a transcript for filtered and unfiltered variants.**

Histogram of the relative position within the coding sequence of unfiltered, putative HLOF variants (left) and after all filters had been applied (right).

**Figure S3. Hardy-Weinberg *p*-values for filtered and unfiltered variants.**

Histogram of the Hardy-Weinberg *p*-value for unfiltered, putative HLOF variants in GS:SFHS (left) and the HLOF variants that had previously been validated (right) (1, 2).

**References**

1. Alsalem, A.B., Halees, A.S., Anazi, S., Alshamekh, S. and Alkuraya, F.S. (2013) Autozygome sequencing expands the horizon of human knockout research and provides novel insights into human phenotypic variation. *PLoS Genet.*, **9**, e1004030.

2. MacArthur, D.G., Balasubramanian, S., Frankish, A., Huang, N., Morris, J., Walter, K., Jostins, L., Habegger, L., Pickrell, J.K., Montgomery, S.B. *et al.* (2012) A systematic survey of loss-of-function variants in human protein-coding genes. *Science*, **335**, 823-828.

3. Kapushesky, M., Adamusiak, T., Burdett, T., Culhane, A., Farne, A., Filippov, A., Holloway, E., Klebanov, A., Kryvych, N., Kurbatova, N. *et al.* (2012) Gene Expression Atlas update--a value-added database of microarray and sequencing-based functional genomics experiments. *Nucleic Acids Res.*, **40**, D1077-1081.

4. Petryszak, R., Burdett, T., Fiorelli, B., Fonseca, N.A., Gonzalez-Porta, M., Hastings, E., Huber, W., Jupp, S., Keays, M., Kryvych, N. *et al.* (2014) Expression Atlas update--a database of gene and transcript expression from microarray- and sequencing-based functional genomics experiments. *Nucleic Acids Res.*, **42**, D926-932.
